# Injectable Thermoresponsive Hydrogels for Cancer Therapy: Challenges and Prospects

**DOI:** 10.3390/gels9050418

**Published:** 2023-05-16

**Authors:** Sandrine Tanga, Marique Aucamp, Poornima Ramburrun

**Affiliations:** 1 School of Pharmacy, Faculty of Natural Sciences, University of the Western Cape, Bellville 7535, South Africa; 3723884@myuwc.ac.za; 2 Wits Advanced Drug Delivery Platform Research Unit, Department of Pharmacy and Pharmacology, School of Therapeutic Sciences, Faculty of Health Sciences, University of the Witwatersrand, Johannesburg 2193, South Africa; poornima.ramburrun@wits.ac.za

**Keywords:** thermoresponsive hydrogels, cancer, injectable hydrogels, chemotherapy, polymers, intratumoral hydrogels

## Abstract

The enervating side effects of chemotherapeutic drugs have necessitated the use of targeted drug delivery in cancer therapy. To that end, thermoresponsive hydrogels have been employed to improve the accumulation and maintenance of drug release at the tumour site. Despite their efficiency, very few thermoresponsive hydrogel-based drugs have undergone clinical trials, and even fewer have received FDA approval for cancer treatment. This review discusses the challenges of designing thermoresponsive hydrogels for cancer treatment and offers suggestions for these challenges as available in the literature. Furthermore, the argument for drug accumulation is challenged by the revelation of structural and functional barriers in tumours that may not support targeted drug release from hydrogels. Other highlights involve the demanding preparation process of thermoresponsive hydrogels, which often involves poor drug loading and difficulties in controlling the lower critical solution temperature and gelation kinetics. Additionally, the shortcomings in the administration process of thermosensitive hydrogels are examined, and special insight into the injectable thermosensitive hydrogels that reached clinical trials for cancer treatment is provided.

## 1. Introduction

Since their introduction in the 1960s, injectable thermoresponsive/thermosensitive hydrogels have been employed for the delivery of chemotherapeutic drugs for cancer therapy. The ability of thermoresponsive hydrogels to remain at the tumour site upon injection has demonstrated their efficiency for targeted therapy and subsequent success over conventional injectable chemotherapeutics. Thermoresponsive hydrogels are able to limit systemic circulation, which causes debilitating side effects such as cardiotoxicity, gastrotoxicity, nephrotoxicity, immunosuppression, and myelosuppression, which stem from the use of intravenously injected chemotherapeutic drugs [1,2,3]. The thermal response of injectable hydrogels relies on the transition of solution to solid/semi-solid at physiological temperature (~37 °C). These hydrogels are often reversible; therefore, temperature changes control their chemical and physical state. Examples of thermoresponsive hydrogels used in cancer therapy include natural polymers, proteins or polypeptides, poly(N-isopropyl acrylamide) (PNIPAM), and poly(ethylene glycol) (PEG)-based block copolymers [4,5,6]. These polymers demonstrate a lower critical solution temperature (LCST) or an upper critical solution temperature (UCST). Below the LCST, the polymers remain in a solution state and become more viscous as the temperature increases. Above the LCST, the polymers undergo a state of gelation. This change from hydrophilic to hydrophobic properties demonstrates the amphiphilic nature of thermoresponsive polymers. Several factors can influence this transition process including the ratio of polymer to hydrophilic components, the dispersion medium, and the concentration of hydrophobic contents [7]. Hydrogels that are formed upon cooling of a polymer solution have an upper critical solution temperature (e.g., gelatin), but this approach may not be suited for intratumoral cancer treatment due to their lack of solidification at normal body temperature and the inability to inject a solid gel. These systems also require high temperatures to prepare the formulation, which may cause instability of the loaded drug and other excipients.

Compared to other stimuli-responsive hydrogels such as photo-, pH-, and radio-sensitive hydrogels, temperature-responsive hydrogels are easier to manipulate, do not need additional reagents or equipment to elicit the desired effect, and have wider applicability [8,9]. These hydrogels simply rely on physiological temperature to undergo physical transition at approximately 37 °C. The enhanced applicability of thermosensitive hydrogels for cancer treatment is evidenced in their rigorous examination of the literature compared to other stimuli-responsive hydrogels. Despite their relevance, thermoresponsive hydrogels present challenges in their design strategy, with drug loading hurdles, deficient degradation, poor mechanical strength, instability, and administration difficulties [10,11,12].

This review discusses the current challenges in the design and use of thermoresponsive hydrogels in cancer therapy and provides an overview of their future prospects. Knowledge in this area will help researchers understand pertinent matters to consider when undertaking research and development in thermoresponsive hydrogels for cancer treatment and thus guide them in forming new strategies to overcome the challenges discussed herein. The unique approach of this review is in its considerations of physiological barriers and the provision of a detailed evaluation of thermoresponsive injectable hydrogels that have been translated to clinical use.

## 2. Physiological Barriers to Drug Delivery in Cancerous Tumours

**Figure 1:**
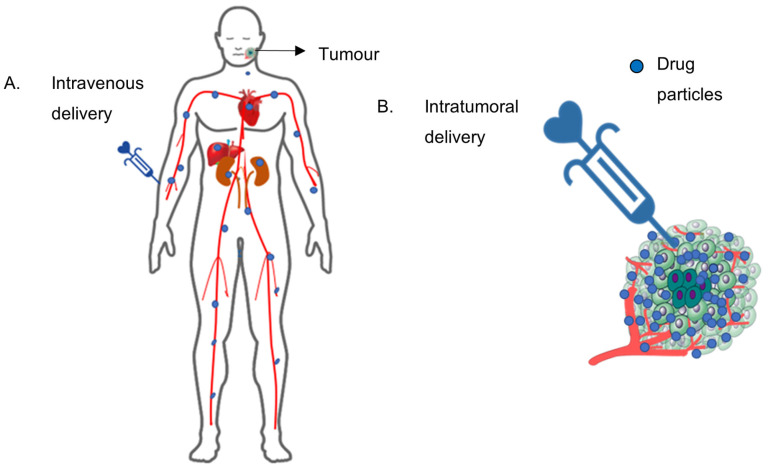
Intravenous delivery and intratumoral delivery. (**A**). Intravenous delivery: the drug is systemically circulated throughout the body. (**B**). Intratumoral delivery: the drug remains localised within the tumour.

Intratumoral delivery provides a significant advantage over intravenous delivery of chemotherapeutics. Direct injection at the site of the tumour can limit systemic circulation and maximise the drug concentration at the target site, thus reducing the side effects of chemotherapeutics and their dosing frequency (Figure 1). Despite these benefits, tumourous tissue has several physical barriers that may obstruct the efficacy of chemotherapeutics by intratumoral delivery from thermoresponsive hydrogels.

**Figure 2:**
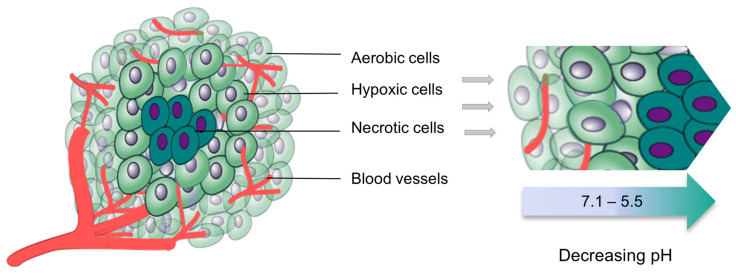
Tumour structure showing hypoxic and acidic variations in different regions.

The tumour is characterised by abnormal vascular growth, a leaky interstitial fluid-filled space with high pressure, and a hypoxic and acidic hostile microenvironment, as depicted in Figure 2. While the interstitial space is advantageous for the accumulation of solidified thermosensitive gels, it can also pose a challenge to drug distribution. Thermosensitive hydrogels with low viscosity and a long gelation time can easily flow out of the tumour and into the systemic circulation via the high-pressure flow in the interstitial space. Additionally, there is a risk of such systems leaking out through the injection site.

The pH of a tumour can range from 5.5–7.1 but the hypoxic environment and acidic variations from the tumour surface to the tumour core may affect drug efficacy at the tumour site [13]. Thermosensitive hydrogels often have a pH-dependent release behaviour with improved release kinetics at lower pH ranges [14]. With the core of a tumour being the most acidic region, thermosensitive hydrogels may demonstrate enhanced performance and drug release kinetics with that acidic microenvironment, yet the core has the least need for chemotherapy due to its necrotic centre. The surface of the tumour is the least acidic region and may be slightly affected with reduced efficacy even though it has the greatest need for chemotherapy since it hosts viable cancer cells. The size of the tumour may also affect the dosing requirements of thermosensitive hydrogels. Selecting the most appropriate dosing regimen may vary based on the agent or combination of agents utilised and the size of the tumour. A larger tumour may need a larger volume of the delivery vehicle so that the whole tumour area is targeted, while smaller tumours or resected tumours may need a smaller volume of the delivery system to avoid unnecessary contact with healthy tissues. Tumours located in areas that endure movements, such as oral cancer and cancerous arthritis located in the joints, require gels that have excellent mechanical strength, elasticity, and adhesion capacity, or else there is a risk of displacing the gel system to non-targeted areas.

Tumours are largely heterogeneous based on their location, size, and functional requisites. However, the physiological challenges of tumours are minor compared to the benefits of drug accumulation promised by thermosensitive hydrogels.

## 3. Selection and Preparation of Injectable Thermosensitive Hydrogels

The design of injectable thermosensitive hydrogels involves careful consideration of the type of polymers and the type of crosslinking method used. Factors such as rheological behaviour and release kinetics are determined by these selections and ultimately influence the behaviour of the thermosensitive hydrogel during preparation and dictate their in vitro and in vivo success.

### 3.1. Physical vs. Chemical Crosslinking

Thermosensitive hydrogels can either be prepared via physical or chemical crosslinking where they undergo sol–gel transition or volume phase transition (VPTT)—swelling in response to temperature. Physical crosslinking entails the linking of one polymer chain to another to produce a thermosensitive effect through non-covalent bonding. These hydrogels undergo electrostatic interactions, hydrophobic interactions, stereocomplexation, and van der Waals forces, which enable them to exhibit a reversible sol–gel response to temperature [15,16,17]. Two key examples are the physical crosslinking of PNIPAM with chitosan and *k*-carageenan by Pourjavadi and colleagues [18] and the crosslinking of PF-127 with gelatin by Yeh and co-workers [19]. Unlike physical crosslinking, chemical crosslinking occurs through the formation of covalent bonds, such as click chemistry, the Michael addition, a Schiff base reaction, photopolymerisation and disulfide bond formation [20,21,22]. Chemically crosslinked hydrogels are not reversible, which may be undesirable for laboratory preparation and clinical use, because once removed from cold storage, the hydrogel must be used, and no further modifications can be made to the formulation. However, the covalent bond formed between the chains of chemically crosslinked polymers enhances their stability in physiological conditions. This stability restricts their dissolution within surrounding fluids, thus limiting their degradation and release rate of drug molecules by diffusion. Physically crosslinked hydrogels suffer poor stability and tunability limitations, whereas hydrogels obtained via chemical crosslinking possess better injectability properties and improved stability [23]. A remarkable study by Han and colleagues portrayed the superior tunability of chemically crosslinked hydrogels. In their study, a chemically crosslinked injectable thermosensitive hydrogel was successfully designed using dialdehyde-functionalised polyethene and β-glycerophosphate crosslinked chitosan for the delivery of intratumoral doxorubicin [23]. Due to Schiff’s reaction, the thermosensitive hydrogels could achieve self-restoration after their destruction and were able to provide sustained release of the drug while maintaining the integrity and function of the hydrogel [23]. The swelling behaviour in physically crosslinked gels is less significant than in chemically crosslinked hydrogels [24]. Consequently, chemically crosslinked hydrogels perform more consistently in vivo and in vitro than physically crosslinked hydrogels. Nonetheless, despite the system accolades of chemically crosslinked thermosensitive hydrogels, the requirement of enzymes, crosslinking agents, and/or organic solvents, has potentially toxic effects; they may undergo cross-reactivity with components of the biological system, damage cells, and denature incorporated bioactive molecules, which may limit the overall application of the injectable hydrogel. Additionally, both physical and thermosensitive hydrogels often swell below their VPTT, which ultimately affects drug release [25]. The VPTT can be modified by including hydrophobic or hydrophilic monomers [25].

### 3.2. Natural vs. Synthetic Hydrogels

Natural polysaccharide polymers, such as chitosan, hyaluronic acid, alginate, and cellulose, have the general advantage of excellent biocompatibility and biodegradability, which makes them preferred candidates for thermoresponsive hydrogel carriers. These polymers are abundant in nature, with good swelling and healing properties. However, their limitation lies in their extremely poor thermal response and therefore restricted applicability. For example, chitosan must be used with glycerophosphate to enhance thermal sensitivity [26,27,28]. This strategy produces a very slow gelation time (~10 min) which may lead to premature drug release upon injection and potential toxicity [29]. If the system is injected and remains a solution for an extended period, it may travel to neighbouring blood vessels outside the tumour and release the drug before it gelates. Chitosan-glycerophosphate produces a fast release of low molecular weight drugs owing to its poor mechanical strength, making it undesirable for chemotherapeutics where long-term drug release is desired [27,29,30]. Moreover, the system’s inability to completely reverse from gel to solution after sol–gel transition was reported by Lu and colleagues [31]. Hyaluronic acid does not favour long-term release as it possesses a very short half-life due to its fast enzymatic degradation by hyaluronidase. Butanediol diglycidyl ether and divinyl sulfone are used to slow the degradation rate of hyaluronic acid [30]. A common attribute between chitosan and hyaluronic acid is that chemical modifications, covalent crosslinking, and gelling agents must be used with these polymers to obtain a gel since they are unable to form hard gels on their own [12,32]. For cellulose, increasing its alkyl groups increases the gelation rate. Methylcellulose, carboxymethyl cellulose, and hydroxypropyl cellulose have shown similar sol–gel behaviour and have been investigated as chemotherapeutic drug carriers [33,34,35]. Interestingly, chitosan and carboxymethyl cellulose have shown dual sensitivity to pH and temperature [36].

The gelation shortcomings of natural hydrogels have necessitated the introduction of synthetic polymers such as PNIPAM and triblock polymers based on polycaprolactone (PCL), poly(d,l-lactide) (PLA), poly(ethylene glycol) (PEG), and poly(amino ester urethane). These polymers can demonstrate rapid thermal response at body temperature and have greater versatility. In contrast to natural thermoresponsive polymers, the greatest weakness of synthetic polymers is their poor biocompatibility and biodegradability. Researchers generally rely on both natural and synthetic polymers for the design of hybrid thermoresponsive hydrogels to obtain desired effects of the biocompatible properties of natural polymers and the tunable properties of synthetic polymers. However, composite hydrogel blends of natural and synthetic polymers may yield viscous gels that are both difficult and painful to inject. There are also concerns about the toxicity of synthetic hydrogel monomers [37]. Extensive work has been conducted to improve the mechanical strength and degradability of synthetic hydrogels while limiting their toxicity. Patenaude and Hoare [38] reported the design of aldehyde-hydrazide-functionalised PNIPAM oligomers with molecular weights below the renal cutoff. The modified PNIPAM was able to degrade over several weeks into non-toxic, low-molecular-weight oligomers. Synthetic-natural thermosensitive hydrogels have reported improved system functions such as mechanical strength and rheological behaviour compared to their individual counterparts [5,39,40].

Additionally, a few studies have explored synthetic hydrogels for gene-based delivery for cancer treatment. However, the main challenge with this delivery is the low transfection efficiency of the hydrogels. The use of cationic polymers which can undergo electrostatic interaction with negatively charged oligonucleotides has shown improved results so far. For example, Dan Zhao and co-workers used PF-127 to design a thermosensitive hydrogel with surviving antisense oligonucleotide and PHB-b-PDMAEMA, which yielded sustained gene release over 16 days and excellent tumour growth suppression in mice of 55% within 4 days [41]. Another study used PEG_550_ -PCL_2200_ -PEG_550_ as a thermosensitive polymer that loaded folate-poly (esteranmine) based on low-molecular-weight polyethyleneimine. The reported transfection efficacy was about 45% and the apoptotic rate was 20% in colon cells [42].

### 3.3. The Drug-Loading Dilemma

Most chemotherapeutic drugs are classified under the biopharmaceutics classification system (BCS) as either class II or IV, with low solubility. A single chemotherapeutic agent is often approved for multiple cancer types—increasing their market demand. For example, paclitaxel (PTX) shows anti-cancer activity against breast, colon, and ovarian cancer, yet it is ranked amongst the lowest soluble chemotherapeutic drugs. Table 1 outlines the poor solubility of chemotherapeutic drugs and their uses in cancer. The extremely low solubility data confirms that there is an increased need for improved delivery of poorly soluble chemotherapeutics since their solubility challenges have severely limited their clinical translation. In contrast, thermoresponsive polymers are mostly soluble in water and their thermoresponsive effect is significantly decreased by the addition of chemotherapeutic drug solvents such as methanol, ethanol, tertiary-butanol, and dimethyl sulfoxide [43,44].

**Figure 3:**
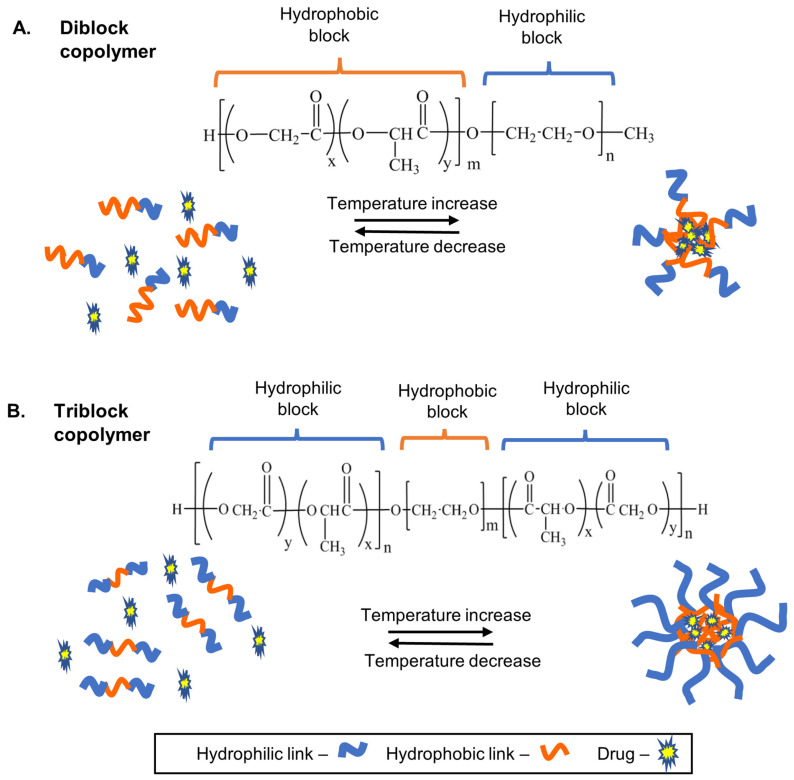
Schematic representation of sol–gel transition mechanisms and hydrophobic drug loading of diblock and triblock copolymers. (**A**) Diblock copolymers (PEG-PLGA) gelate at higher temperatures with hydrophobic drugs bonded at hydrophobic core. (**B**) Triblock copolymers (PLGA-PEG-PLGA) gelate at increased temperatures with more hydrophilic linkages and less hydrophobic ends.

Three common types of thermosensitive hydrogels are often employed for thermosensitive hydrogel constructs: diblock copolymers such as poly(ethylene glycol)-b-poly(D, L-lactide-co-glycolide) (PEG-b-PLGA), triblock copolymers such as poloxamers, and PNIPAM. Diblock copolymers are generally composed of a hydrophilic PEG block and a hydrophobic attachment, for example, methoxy poly(ethylene glycol)-poly-ε-caprolactone (MPEG–PCL) [45]. The PEG component introduces compatibility and controls the drug release, while the hydrophobic segment can introduce biodegradability and mediate the encapsulation of hydrophobic drugs. Although the presence of the hydrophilic moiety is essential for sol–gel transition, it also contributes to poor drug loading of hydrophobic chemotherapeutics. This concept is further exaggerated in triblock copolymers, which contain a hydrophobic A-block and a hydrophilic B-block unit. Poloxamers such as poloxamer 407, 188, and 388 are made of only one poly(propylene oxide) group and two hydrophilic blocks [46]; thus, the loading of hydrophobic chemotherapeutics into poloxamer-based hydrogels is therefore severely limited. For example, Carrillo-Castillo and colleagues prepared a thermoresponsive PCL–PEG–PCL triblock copolymer which loaded only 1 ug/mL of methotrexate with no significant improvement to the drug’s aqueous solubility [47]. Insoluble drugs may also become heterogeneously distributed within the hydrogels, leading to variabilities and non-uniformity of drug release rates from hydrogel samples. Consequently, hydrogel matrices with hydrophilic end blocks exhibit poor solubility, limited drug-carrying capacity, and a minimal ability to sustain drug release for prolonged periods. Figure 3 depicts the sol–gel transition and drug loading of diblock and triblock copolymers. Attempts to improve this deficiency in amphiphilic thermosensitive hydrogels have been explored by combining block copolymers of differing molecular weights and ratios [47], including complexes such as cyclodextrins (CD) [48] or synthesising dual delivery systems such as the use of nanocarriers and liposomes, with targets for thermoresponsive sol–gel properties [49,50,51]. One study maximised this hybrid strategy by loading nanocrystals into a thermosensitive hydrogel constructed with poloxamer 407, poloxamer 188, and carbomer 974P to generate PTX-nanocrystalline gel [52]. The researchers reported an increased loading of PTX at 10 mg/mL with adequate rheological behaviour and the prevention of local tumour recurrence in mice [52]. In another study, when PTX was loaded in nanocrystals and niclosamide nanocrystals with PLGA-PEG-PLGA thermosensitive hydrogel, its loading capacity was increased to 4 mg [53]. These reflect the importance of dual delivery systems to aid thermosensitive hydrogel drug delivery.

**Table 1:** Solubility of chemotherapeutics and their examples in thermosensitive hydrogel systems.

Drug	Type of Cancer Commonly Indicated for	Solubility in Aqueous Solution (mg/mL)	Examples of Thermoresponsive Delivery Systems	Reference
Cisplatin	Prostate, ovarian, and bladder cancer	~1	Co-delivery of resveratrol microspheres and cisplatin into pluronic-F127 hydrogel against H22 cells.	[54,55]
Paclitaxel	Breast, colon, and recurrent ovarian cancer	~0.002	Paclitaxel nanocrystals loaded into poloxamer 407, poloxamer 188, and carbomer 974P against breast cancer.	[52,56,57]
Doxorubicin	Leukemia, breast cancer, soft tissue and bone sarcoma, ovarian, bladder, thyroid, and gastric carcinoma	<10	Co-delivery of doxorubicin and cisplatin loaded in PLGA-PEG-PLGA hydrogel against Saos-2 and MG-63 cells.	[58,59]
Docetaxel	Prostate cancer, metastatic breast cancer, and gastric cancer	0.006–0.007	Black phosphorus nanosheets and micelle docetaxel loaded in PF-127 thermoreversible hydrogel for chemo-photodynamic therapy.	[60,61]
Daunorubicin	Leukemia	~0.3	-	[62]
Tamoxifen	Breast Cancer	~0.0003	Tamoxifen nanoparticles loaded in PLGA-PEG-PLGA against MCF-7 cells in breast cancer.	[63,64]
Etoposide	Testicular, prostate, bladder, stomach, and lung cancer	∼0.008	Etoposide loaded in poloxamer 407/poloxamer 188 thermosensitive hydrogel for sustained drug release.	[65,66,67]
Irinotecan	Colorectal cancer	~0.107	Irinotecan-loaded solid lipid nanoparticles in a poloxamer 407/polaxamer 188 thermosensitive hydrogel for colorectal cancer.	[68]
5-fluorouracil	Breast, colorectal, stomach, and pancreatic cancer	12	5-fluorouracil loaded into polaxamer 407/polaxamer 188/alginate thermosensitive hydrogel for colorectal cancer.	[69]
Methotrexate	Non-Hodgkin’s lymphoma, breast, ovarian, and lung cancer, and epidermal tumors of the head and neck.	~0.067	Methotrexate carbon nanotubes were loaded into a chitosan/β-glycerophosphate thermosensitive hydrogel.	[70]
Bleomycin	Squamous cell carcinoma of the head and neck, testicular carcinoma, Hodgkin lymphoma	20	Bleomycin liposomes loaded in PF-127/PF-68 thermosensitive hydrogel.	[71]

### 3.4. Lower Critical Solution Temperature

For a thermosensitive hydrogel system to excel in its intended application, gelation must occur above room temperature but below body temperature, i.e., 26–36 °C. However, achieving an LCST within the narrow range of 26–36 °C is challenging. If the gelation temperature of the injectable gel is below 26 °C, gelation occurs at room temperature, leading to difficulties in manufacturing and handling. The premature gelation also risks needle clogging and consequent administration difficulties, which are elaborated on further in this review. Additionally, high LCSTs produced through physical crosslinking are usually accompanied by low mechanical strength at physiological temperatures [72,73]. Many thermoresponsive polymers are restricted in their use due to their high LCSTs as presented in Table 2. However, during hydrogel preparation and drug loading, the introduction of crosslinkers, manipulations in material concentrations, polymer ratio [7], and the type of dispersion media used play a substantial role in determining the LCST of the designed hydrogel carrier. The LCST can also be manipulated by changing the copolymer block length. Increasing the block length increases the aggregation tendency of the copolymer in water, resulting in a lower LCST and quick onset of gelation at a lower concentration [73,74,75]. For PEG-based amphiphilic copolymers, a lengthy polyester block, shorter PEG block, and increased hydrophobicity or crystallizability of the polyester block lead to a lower LCST. In general, increasing the ratio of hydrophobic groups results in a low LCST, while increasing the ratio of hydrophilic groups produces a higher LCST [76]. Additionally, the LCST can be tuned via polymer grafting wherein the end chain of the thermosensitive polymer is modified using hydrophobic or hydrophilic moieties. For example, Konstantinos et al. grafted alginate backbone to various concentrations of N-isopropylacrylamide/N-tert-butylacrylamide random copolymer (PNIPAMx-co-NtBAMy) in order to decrease the LCST. The LCST shifted from 38 to 20 °C by enriching the PNIPAM chains with 20 mol % NtBAM [77]. In another study by Fang and co-workers, chitosan was grafted onto PNIPAM and loaded with cisplatin [78]. The drug-loading capacity of cisplatin in the thermosensitive hydrogel increased from 42% to 72% after polymer grafting [78].

### 3.5. Dynamics of Drug Release

Thermosensitive hydrogels are controlled by either erosion, swelling, or diffusion-based drug release mechanisms [87]. In the erosion mechanism, the drug is depleted from the surface of the hydrogel as it dissolves in aqueous media. In swelling-controlled drug release, the hydrogel absorbs water, leading to an expansion of the hydrogel pores and subsequent release of the drug. Diffusion-controlled hydrogels rely on drug movement from an area of high concentration to a low concentration according to a concentration gradient.

**Figure 4:**
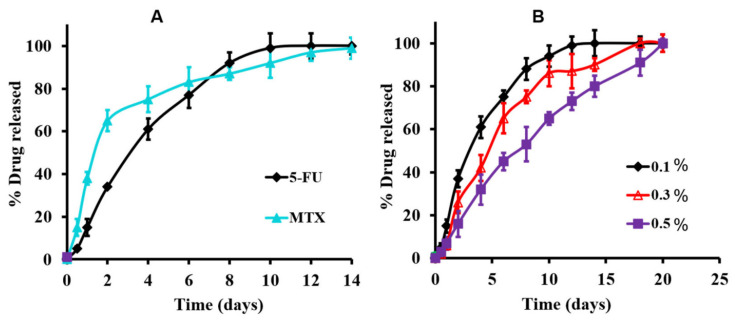
(**A**) In vitro release profiles of the individual 5-fluorouracil and methotrexate from the modified hydrogel at 37 °C in PBS at 0.1% drug concentration, and (**B**) the release profile of 5-fluorouracil as a function of drug concentration in PBS at 37 °C [97]. Reproduced with permission from Almawash, El Hamd, and Osman 2022 © Creative Commons CC BY 4.0.

The drug release mechanism of chemotherapeutics through the hydrogel matrix is impacted by various factors including hydrophobicity, mechanical strength, pore size, and degradation rate. Synthetic thermoresponsive hydrogels demonstrate the rapid release of hydrophobic chemotherapeutic drugs and struggle to achieve prolonged, on-demand, and rhythmic drug delivery [14,55,88]. Moreover, the contrast of hydrophobic drugs in hydrophilic systems often generates a fast release rate and is characterised by an initial burst effect [89]. The initial burst release of thermosensitive hydrogels usually ranges between 20–60% within the first day [53,70]. Burst release can compromise the intended drug dosage and cause tissue toxicity. A synthetic hydrogel such as PNIPAM is characterised by hydrophilic amide and hydrophobic propyl groups. Below its LCST, its polymer chains extend due to hydrogen bonding between the amide groups and the water molecules. Increasing the temperature weakens the hydrogen bonds between the amide and water molecules and increases the hydrogen bonding between hydrophobic interactions among the propyl groups [90]. However, PNIPAM-based hydrogels’ separation from solvent and shrinkage above the LCST may permit the uncontrolled release of drug molecules [91,92]. This poor release behaviour is also true for pluronics which encounter burst release due to their low molecular weight and mechanical strength. The many hydrogen bonds between thermosensitive polymer chains form a relatively loose and porous three-dimensional network that allows drug molecules to easily diffuse out of the gel matrix [93]. Resolutions to this challenge are the use of hydrophobic moieties or the addition of complexing agents such as CDs that allow the hydrogel system to be homogeneously incorporated with the hydrophobic drug [76,92,94,95,96]. Fiorica et al. [89] designed an injectable hydrogel by using hyaluronic acid with vinyl sulfone-functionalised β-CDs as a crosslinking agent to obtain a thermal response. Doxorubicin was loaded into the system and investigated for its use in locoregional tumours. The system maintained a sustained release of doxorubicin when tested in colorectal carcinoma micro masses. Recently, an inclusion complexation between polymerised β-CD and hydrophobic cholesterol end-capping polyethylene glycol, loaded with 5-fluorouracil/methotrexate was constructed as a thermoresponsive hydrogel [97]. The researchers stated that the benefit of polymerised β-CD, cholesterol end-capping PEG in cancer delivery, had not been fully reported yet. Therefore, they sought to provide in vitro and in vivo application of the modified hydrogel in breast cancer management for the first time. Figure 4 shows the in vitro release profiles obtained for the two anti-cancer drugs (5-fluorouracil/methotrexate), which revealed extended-release behaviour of up to 21 days. The novel study demonstrates the impact of the newly assembled hydrogel for controlled release and efficient delivery of the two loaded drugs. These studies also emphasise the importance of CDs in controlling the release behaviour of thermosensitive hydrogels due to their hydrophilic surface and hydrophobic core, which helps to encapsulate hydrophobic drugs.

**Figure 5:**
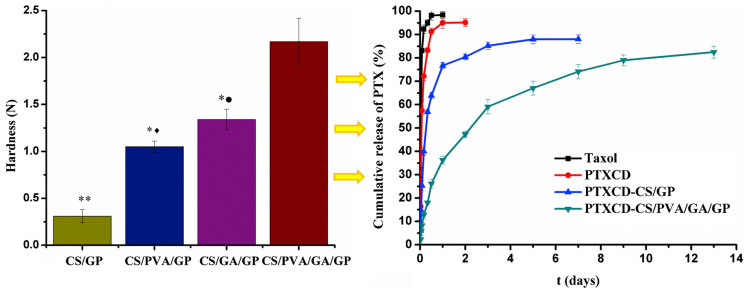
Drug release-dependent behaviour on mechanical strength for PTX-CD loaded hydrogels (^*●^ *p* < 0.01, CS/PVA/GA/GP vs. CS/PVA/GP, ^*♦^ *p* < 0.01 and CS/PVA/GA/GP vs. CS/GP, ^**^ *p* < 0.01) [93]. Reproduced with permission from Elsevier B.V. Ltd. © 2016.

The mechanical strength of hydrogels also plays a pivotal role in determining drug release rates and researchers rely on compressive strength or storage modulus to ascertain the strength of the hydrogel. Hard gels release chemotherapeutics at a slower rate, while softer gels release chemotherapeutics faster [8,9]. As the gel hardens, the degree of porosity decreases, restricting the flow of water and drugs out of the hydrogel matrix. Sustained drug release is beneficial in cancer treatment because chemotherapeutics often require a long treatment period. Achieving this slow drug release rate through the mechanical enhancement of injectable hydrogels is difficult. While most synthetic thermosensitive systems promote rapid gelation, their formed gel severely lacks strength [98]. This demands the need for a greater concentration of the polymer or inclusion of crosslinkers in the hydrogel system [99,100,101,102,103] which affects rheology, restricting the flow of the liquid at cooler temperatures and decreasing the LCST. Additionally, increasing synthetic polymer concentration makes the gel hard but brittle, leading to breakage of the hydrogel system and, ultimately, unsteady drug release [104]. Jiang et al. [93] compared the drug release behaviour of PTX-CD loaded in chitosan (CS)/glycerol phosphate disodium salt (GP) and CS/PVA/glutaraldehyde (GA)/GP. Figure 5 shows that the mechanical strength of CS/GP was lower than that of CS/PVA/GA/GP, and, consequently, the release time of PTX increased as the hardness of the hydrogel increased. Therefore, the strength and elasticity of the system hold great importance wherein the matrix remains accumulated in one area and maintains its shape within the tumour structure while retaining the drug for a prolonged period. There are no standards for the recommended mechanical strength of thermosensitive hydrogels and results to this effect vary in the literature including the related methodologies to obtain these values. However, it can be presumed that the mechanical strength should aim to mimic that of the extracellular matrix of the targeted area. Recently, a range of storage modulus between 1000–16,000 Pa, at physiological temperature, has been reported as having good mechanical strength for sustained drug release [51,69,104,105].

The release of chemotherapeutics is substantially influenced by the degradation rate of the thermosensitive hydrogel. This rate depends on the polymer composition, crystallinity, and topology. The issue of hydrogel degradation is of concern because most synthetic polymers possess poor biodegradation properties with rates that do not correspond to the dosing frequency of the loaded chemotherapeutic drug. Additionally, synthetic polymers such as PEG/polyesters produce acidic molecules that may impair drug performance [106] or promote inflammatory responses by host tissues. Their degradation time can also be very long [107], thus impeding the transport of drug molecules to the tumour via an erosion-based release mechanism. The chemical structure of the thermosensitive hydrogel [108], the inclusion of natural components such as citric acid [109], or even the use of natural polymers can be exploited to develop hydrogels with tailored degradation rates and drug release behaviour. Amongst synthetic polymers, poly(lactic-co-glycolic acid) (PLGA)-based hydrogels have reported the best degradation results for thermosensitive hydrogels [110,111]. With the improvements in thermosensitive hydrogel designs, recent studies have reported drug release from 60–100% within 1 month [102,112,113].

### 3.6. Physical Mixing Hurdles

Another challenge with preparing injectable hydrogels is the large quantity requirement of the polymer concentration needed to achieve a thermal response at 37 °C, particularly for triblock copolymers [5,114]. This is caused by the polymer’s high critical micelle concentration due to weak bonds with the hydrophobic blocks. Studies report that at least 15–30% pluronic-F127 (PF-127) [55,115,116], 20% poly(l-lactide-*co*-glycolide)-poly(ethylene glycol)-poly(l-lactide-*co*-glycolide) (PLGA–PEG–PLGA) [117,118], and 10% poly(organophosphazene) (PPZ) [119] are required to obtain a thermal response at physiological conditions. Hydrogels with LCST around 25 °C may also pose mixing challenges due to their high viscosity at ambient temperature. This further demands cold mixing, which may be difficult to control and maintain during the fabrication process. PEG/PLGA and polyphosphazene polymers have a paste-like consistency which hampers weighing and poses mixing challenges with the drug and other excipients. Poly(ε-caprolactone)-poly(ethylene glycol)-poly(ε-caprolactone) (PCL-PEG-PCL) has a strong crystallinity and therefore needs to be dissolved in an aqueous solution at a temperature above its melting point (60 °C). Thereafter, the polymer must be incubated at 4 °C for 24 h to obtain a thermosensitive hydrogel—a multi-step preparation process that may limit its large-scale manufacturing and clinical translation.

## 4. Administration of Injectable Thermosensitive Hydrogels

The administration process of thermosensitive hydrogels may pose several challenges to patients and clinicians. Syringeability and injectability are important factors that must be considered during the design of thermosensitive hydrogels. The system should be able to flow freely through the injection needle at ambient temperature, and should easily be injectable at the target site. An injection force of less than 40 N for subcutaneous administration is acceptable with a maximum needle gauge of 25 G [120,121]. In an attempt to reach optimal gelation properties, such as improved mechanical strength, the concentration of polymer is often increased [122]. Additionally, the high critical micelle concentrations of thermosensitive polymers necessitate a high concentration of copolymers to obtain gelation [123,124], leading to a more viscous solution below the LCST. This increase in viscosity warrants the need for a large injection needle that is inserted at the tumour site, allowing the viscous hydrogel solution to flow and press against the extravascular tumour tissue as it fills the space. For the clinician, a high injection force is required and for the patient, it is a painful experience and undoubtedly affects their determination to allow repeated treatment at the tumour site. However, the use of a local anaesthetic may help prevent this scenario. If the thermosensitive gel has a low LCST, gelation of the hydrogel will occur at room temperature, causing the system to become clogged in the needle and therefore wasted. Clinicians would have to hasten administering the injection to ensure that the gel is removed from cold storage and injected within a limited time frame before the system gels. This may be an even greater concern in undeveloped and developing tropical countries where ambient temperatures are often elevated, and there is a lack of reliable cooling systems.

Another regretful administration challenge with injectable hydrogels is that they are inaccessible to internal organ cancers such as liver, pancreatic, and oesophageal cancer. If a need for the system is established in such a case, the use of specialised techniques such as endoscopic ultrasonography-guided fine-needle injection is required to allow direct access to the tissues. Surgery is employed for inaccessible sites such as brain and ovarian cancers. These processes require a specialist medical professional, and this can be costly and inconvenient for the patient. Metastatic cancers are also non-benefactors of injectable thermosensitive systems since they only localise at the injected tumour site. In this case, the hailed benefit of thermosensitive drug accumulation reaches a limitation. Additionally, for a thermosensitive hydrogel to be employed, imaging techniques must precisely identify the cancer tumour, or else there is a risk of treating one tumour site while another area, unidentified by imaging, is left untreated.

## 5. Thermoresponsive Hydrogels in Clinical Trials: An Update

To date, clinical trials on injectable thermosensitive hydrogels for cancer treatment have been severely limited (Table 3). UroGen Pharma commercialised ReGel^®^, a (PLGA-PEG-PLGA)-based formulation that can undergo sol–gel transition at 37 °C. The strength of the system is in its ability to solubilise poorly soluble drugs [53,125], and based on this advantage, OncoGel^®^ was fabricated. OncoGel^®^ is a chromophore-free, paclitaxel formulation of triblock copolymer (PLGA–PEG–PLGA) (ReGel^®^) intended for local tumour management [126]. It increases paclitaxel drug loading by more than 400 fold (>10 mg/mL) and reports excellent release results and degradation over 4–6 weeks [127]. OncoGel^®^ is currently the only existing injectable thermosensitive hydrogel that has undergone clinical trials for cancer treatment [128]. Despite efforts to make OncoGel^®^ applicable, its phase 2 clinical trial was terminated. The researchers noted that, although safe, overall survival or tumour response remained the same when the gel was used with cisplatin/5-fluorouracil and radiation therapy in patients with previously untreated, resectable, local or local–regional adenosarcoma or squamous cell carcinoma [129]. A dose escalation study of OncoGel^®^ was unsuccessful in another phase 2 clinical trial when evaluated within a tumour resection cavity in the brain following surgical removal of the tumour [130].

Although not for direct injection, UGN-101 (Jelymyto^®^) was constructed with thermoresponsive sol–gel properties for the delivery of mitomycin in upper tract urothelial carcinoma [131]. The system is based on ReGel^®^ and has an LCST around body temperature [132]. UGN-101 overcomes the physio-anatomical constraints of the urinary tract, where continuous urine production prevents drug retention [132]. After favourable phase 3 clinical trials, which revealed significant disease eradication and reduced nephrotoxicity [133], the system received FDA approval under the orphan drug designation [134]. Jelymyto^®^ has since been registered for a clinical trial to assess its efficacy and safety in recurrent patients who already received the drug for upper tract urothelial carcinoma; however, the study was withdrawn due to a lack of participants, owing to the rarity of the disease [135]. Another clinical trial was recently completed for the thermosensitive mitomycin in nonsurgical primary chemoablation of nonmuscle invasive bladder cancer [136,137]. The system remained durable and achieved significant recovery with no reoccurrence within one year in 65% of the patients [136].

The clinical trials presented herein validate the safety and relevance of thermosensitive hydrogels in cancer treatment. The failed studies do not prove that thermosensitive hydrogels are ineffective, but rather that more hydrogels with improved release qualities should be designed and various systems should be investigated for different cancer types. The lesson is also to look beyond the concept of intratumoral delivery and propose specialised methods to deliver thermosensitive hydrogels for various cancer types, as Jelymyto^®^ has demonstrated.

**Table 3:** Thermosensitive hydrogels that have undergone clinical trials for cancer treatment.

Trade Name	Encapsulated Drug	Thermosensitive Hydrogel	Cancer Type	Status	References
OncoGel^®^	Paclitaxel	PLGA-PEG-PLGA	Esophageal cancer; adenocarcinoma of the esophagus; squamous cell carcinoma; brain neoplasms;	Phase 2	[126,128]
			glioblastoma multiforme	Phase 2	[131]
Jelymyto^®^	Mytomycin	PLGA-PEG-PLGA	Carcinoma; transitional cell; transitional cell; carcinoma of renal pelvis;	Phase 3	[131,133]
			bladder cancer	Phase 2	[136]

## 6. Conclusions and Future Outlook

Injectable thermosensitive hydrogels are currently a popular research venture; however, there is a significant lack in their clinical translation. The challenges of tumour structural barriers, the poor loading of chemotherapeutics, unsustained drug release, and inefficient gel rheology have limited their clinical efficacy. However, their promise for targeted drug delivery at the tumour site remains the backbone of their rigorous laboratory examination. Synthetic polymers have paved the way for the enhancement and tunability of injectable thermosensitive hydrogels, but qualities of poor biocompatibility and degradability restrict their successful implementation. Natural polymers or additives for the promotion of these limitations are encouraged for use with synthetic thermosensitive hydrogels and the balancing of hydrophilic and hydrophobic components is an integral part of sol–gel behaviour. Hybrid systems such as the use of nanocarriers, extravesicular carriers, and inclusion complexes such as cyclodextrins can tackle poor drug loading of thermosensitive hydrogels. Furthermore, the heterogeneity of different tumour types emphasises the need for personalised treatment plans.

A critical need for improved systems in the field of thermosensitive hydrogels still exists, and future research should focus on improving current or designing new synthetic polymers for the increased drug loading of hydrophobic chemotherapeutics while maintaining good mechanical strength, sol–gel transition, and biodegradable and biocompatible properties. Additional anti-cancer drugs, such as dostarlimab (recently approved for endometrial cancer), should be explored for thermosensitive hydrogel formulation. Researchers should also consider the repurposing of drug agents for thermosensitive hydrogel designs, such as aspirin and terbinafine, which have shown efficacy toward cancer cells [138,139]. Thermosensitive hydrogels still have a long way to go in the field of advanced drug delivery. As research advances in the field of stimuli-responsive hydrogels to overcome the associated challenges, it is anticipated that more thermosensitive systems will be studied in clinical trials.

## Figures and Tables

**Table 2:** Lower critical solution temperature of commonly used thermosensitive polymers and polymer blends in water.

Polymer	Polymer Concentration in Aqueous Solution (% *w*/*v*)	LCST (°C)	Reference
Poly(N-isopropyl acrylamide), PNIPAM	~2.5	~32	[79]
Poly(vinyl methyl ether), PVME	~5	~40	[80]
PLGA-PEG-PLGA	~25	~25	[81]
Poly(*N*-vinylcaprolactam), PNVCL	~0.5	~30	[82]
Chitosan–glycerol phosphate	~1 CH + ~10 GP	~37	[83]
Pluronic-F127, PF-127	~15	~25	-
Hydroxypropyl methylcellulose, HPMC	~1	~70	[84]
Polyphosphazene derivatives	~2	25–80	[85]
Methoxy poly(ethylene glycol) (MPEG)–diblock copolymers)	~1	32–42	[86]

## Data Availability

Not applicable.
